# Integrated Low-Loss, High-Isolation, and Broadband Magneto-Optical Isolator with TE-Mode Input

**DOI:** 10.3390/mi16030315

**Published:** 2025-03-09

**Authors:** Li Liu, Jia Zhao, Chen Zhang

**Affiliations:** School of Information Science and Engineering, Shandong University, Qingdao 266237, China; liulisrzh@163.com (L.L.); zhaojia@sdu.edu.cn (J.Z.)

**Keywords:** magneto-optical isolator, non-reciprocal phase shift, TE mode, AlGaAs-on-insulator, figure of merit

## Abstract

High-performance optical isolators are key components in photonic integrated circuits, with significant applications in nonlinear optical systems. We propose a design for a TE-mode optical isolator based on the AlGaAs-on-insulator platform. The isolator consists of non-reciprocal phase shift (NRPS) waveguides, reciprocal phase shift (RPS) waveguides, and multi-mode interference (MMI) couplers achieving low loss, high isolation, and wide bandwidth. Numerical simulations show that, at a wavelength of 1550 nm, the device provides a bandwidth of 91 nm at 30 dB isolation. The confinement factors for a magneto-optical (MO) waveguide were analyzed, and a detailed loss analysis revealed a total loss of 1.47 dB and a figure of merit (FoM) of 2.76 rad/dB. The manufacturing tolerances of the isolator are discussed referring to the requirement of stability and reliability in practical applications. This study provides an optimized design for high-performance TE-mode optical isolators in integrated photonic systems, which are well-suited for efficient and stable nonlinear optical applications.

## 1. Introduction

For decades, aluminum gallium arsenide (AlGaAs) has become one of the most widely used materials in nonlinear photonics, primarily due to its large electro-optic effect (34.8 pm/V) [1], high second-order nonlinear coefficient (40 pm/V) [2], and a wide transparent window from near-infrared to mid-infrared (0.9–17 µm) [3,4]. These nonlinear optical properties of AlGaAs provide significant advantages in applications of integrated photonic circuits, including all-optical signal processing [5,6,7] and integrated quantum photonics [8]. The integrated AlGaAs photonic devices not only have miniaturation but also enable both passive and active functions on the same chip. This reduces the footprint of the optical system and, due to the higher photon density [9], enables modulation and wavelength conversion at lower power levels.

In the field of integrated photonics, silicon (Si) photonics shows the greatest application potential in the commercial market [10,11]. Optical isolators, as a key component of photonic integrated circuits (PICs), precisely control the transmission of unidirectional light and prevent reflected light from damaging the active devices [12,13,14]. Magneto-optical (MO) MZI-type and ring-type isolators based on silicon-on-insulator (SOI) platforms have been reported [15,16,17,18,19], exhibiting high isolation (25 dB) [17] and low insertion loss (4 dB) [19]. However, waveguide materials, such as Si and silicon dioxide (SiO_2_), with centrosymmetric crystal structures or amorphous properties, have significant two-photon absorption (*α*_2_) and free-carrier absorption, which is detrimental for their nonlinear optical effect. Moreover, as indirect bandgap materials, they are generally inefficient for light-emitting applications. Therefore, there are still limitations in achieving fully integrated nonlinear photonic circuits on SOI. Various AlGaAs-based devices have been realized on insulator platforms, such as micro-ring resonators [20,21], chip-based frequency combs [22,23], and wavelength converters [24,25]. Recently, Lin et al. [26] pointed out that the propagation loss coefficient of the AlGaAs on an insulator waveguide is approximately 0.4 dB/cm, comparable to the loss of SOI waveguides. These properties provide great potential for the development of AlGaAs-integrated nonlinear functional monolithic circuits.

Currently, most research on waveguide-type MO isolators mainly focuses on the TM mode, and our prior work has also proposed TM-mode-based magneto-optical isolators using GaAs-on-insulators [27]. However, since the output of most semiconductor lasers is TE polarization, TE-mode isolators are indispensable in many applications. To maximize the nonreciprocal polarization rotation (NRPS) in the TE polarization, the magnetic material should be placed on the side of the waveguide (which can only be achieved by deposition techniques), rather than above the waveguide as in traditional TM-mode isolators, which typically requires wafer bonding or deposition techniques. Although both GaAs and AlGaAs have advantages, such as a wide transparent window, high refractive index, and low loss, AlGaAs generally has more advantages than GaAs in MO isolator applications. By adjusting the Al content (*x*), the bandgap energy of Al*_x_*Ga_1-*x*_As can be precisely controlled. For example, in the telecom C-band, the bandgap of Al_0.18_Ga_0.82_As blue-shifts significantly, which reduces *α*_2_ while maintaining a high refractive index (*n*_0_ > 3), thus optimizing the isolator’s transmission loss [28]. In contrast, pure GaAs has a higher α_2_, requiring additional compensating structures. Additionally, lowering the Kerr coefficient by adjusting the *x* value enhances the isolator’s stability and reduces thermal effects [29]. Finally, by adjusting the Al content, the lattice constants of different materials can be precisely matched, and during the epitaxial growth process, defects are typically minimized, especially when integrating with other III–V semiconductor materials, such as InP, which is crucial for monolithic integration [30].

Here, we describe a MO isolator based on an asymmetric MZI structure with TE input on an AlGaAs-on-insulator platform. The isolator achieves low loss, high isolation, and wide bandwidth, and is suitable for integrated nonlinear photonic circuits. The structure includes two multi-mode interference (MMI) couplers, NRPS waveguides, and reciprocal phase shift (RPS) waveguides, with the MO waveguide composed of AlGaAs/YIG/Ce:YIG thin films. Using the full-vector finite element method (FEM), we optimized the structural parameters of the MO waveguide and calculated the variations in the NRPS for different waveguide thicknesses and widths. With an NRPS of 2332.62 rad/m and a waveguide length of 673.40 µm, the MMI coupler and RPS waveguide were designed based on the optimized MO waveguide parameters. The input and output structures of the MMI coupler were optimized using the eigenmode expansion (EME) method. We used the finite difference eigenmode (FDE) solver to explore the relationship between bend radius and loss, and conducted a detailed analysis of the losses associated with the coupler, coupling waveguide, and MO waveguide. Ultimately, the overall loss of the device was only 1.47 dB. Additionally, the figure of merit (FoM) of the device was calculated. Finally, the relationships between geometric parameters, isolation bandwidth, and manufacturing tolerances were discussed.

## 2. Proposed Device and Principle

**Figure 1:**
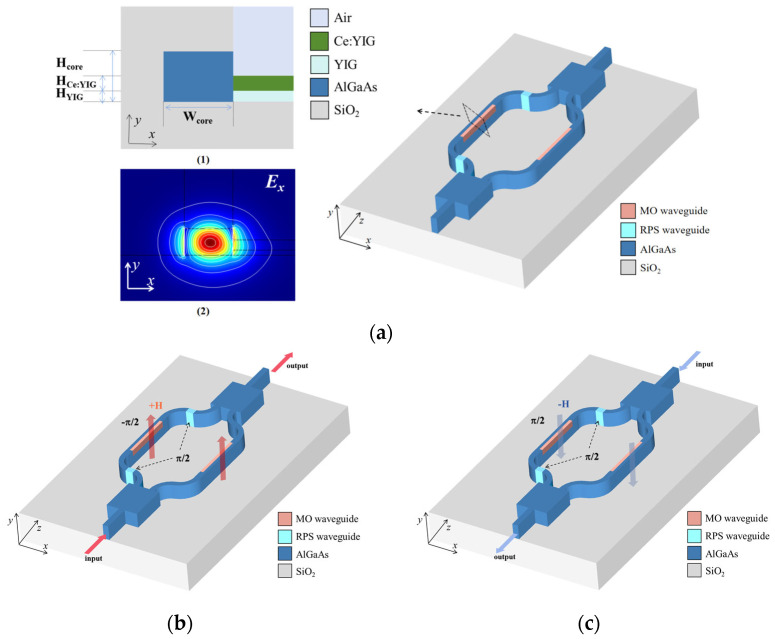
(**a**) Schematic diagram of magneto-optic isolator on AlGaAs-on-Insulator. (**1**) Cross-section of MO waveguide and (**2**) *E_x_* field distribution of TE mode. (**b**) When magneto-optic waveguide is magnetized in +*x*-direction, propagation constant is in the opposite direction. (**c**) When magneto-optic waveguide is magnetized in −*x*-direction, propagation constant is in the opposite direction.

The MZI-type optical isolator operates in a single-polarization mode, successfully overcoming the phase-matching issue in the TE-TM modes. The device uses an asymmetric MZI structure, as shown in Figure 1. At both ends of the device, 3 dB 1 × 2 MMI couplers are configured. The upper arm consists of the MO waveguide and RPS waveguide, while the lower arm is made of the MO waveguide. (1) in Figure 1a shows the cross-sectional structure of the MO waveguide. In the *x*-direction, the NRPS effect is generated by depositing Ce:YIG/YIG on the side of the core layer of AlGaAs, with the substrate being SiO_2_. The typical refractive indices of the layers are *n*_SiO2_ = 1.45, *n*_AlGaAs_ = 3.2, and *n*_Ce:YIG/YIG_ = 2.2. Notably, the refractive index contrast between the core layer and the substrate reaches 0.397, which effectively confines the light field and ensures the stability and robustness of the transverse optical modes.

The magneto-optic effect can be described by the off-diagonal elements of the material’s dielectric tensor. In the waveguide, the TE mode propagates along the *z*-direction. When the MO material is magnetized in the *y*-direction, the dielectric constant tensor of the material can be expressed as follows:

(1) $$\varepsilon_{\pm }=\left[\begin{array}{ccc} \varepsilon_{xx} & 0 & \pm j\varepsilon_{xz}\\ 0 & \varepsilon_{yy} & 0\\ \mp j\varepsilon_{xz} & 0 & \varepsilon_{zz} \end{array}\right]$$

where $\varepsilon_{xx}=\varepsilon_{yy}=\varepsilon_{zz}=n_{Ce:YIG/YIG}^{2}$ are the diagonal elements, representing the permittivity in different directions, and $\varepsilon_{xz}$ is the off-diagonal element, which represents the strength of the magneto-optic effect. The key to the MO effect lies in the introduction of material anisotropy by the off-diagonal elements under the influence of an external magnetic field, leading to changes in the polarization and propagation characteristics of light. The expression for $\varepsilon_{xz}$ is as follows:

(2) $$\varepsilon_{xz}=\frac{n_{Ce:YIG/YIG}\theta_{F}\lambda }{\pi }$$

where *θ_F_* is the Faraday rotation coefficient and λ is the wavelength of light. In the simulation, the *θ_F_* value of Ce:YIG at a wavelength of 1550 nm is set to −5900 deg/cm [31], while YIG is 200 deg/cm [32]. The corresponding $\varepsilon_{xz}$ are calculated to be 1.12 × 10^−2^ and 3.79 × 10^−4^.

The single-mode waveguide excites the fundamental TE mode (TE_0_) in the waveguide region, and due to the overlap of this mode with the Ce:YIG/YIG cladding, they exhibit a significant NRPS in the propagation constants. When the TE_0_ mode propagates along the *z*-axis in the MO waveguide, the forward and backward propagation constants (*β_f_* and *β_b_*) differ in the two propagation directions. Manipulating the direction of the light propagation or the direction of the static magnetic field causes changes in these two constants. NRPS is defined as the difference between these two propagation constants, and it is described using perturbation theory [33]:

(3) $$\begin{array}{c} ∆\beta =\beta_{f}-\beta_{b}=\frac{2\omega \varepsilon_{0}}{\beta P}\iint \varepsilon_{xz}E_{x}\partial_{x}E_{x}dxdy\\ {∆\varphi }_{NRPS}=∆\beta \times L_{NRPS} \end{array}$$

where *ω* represents the frequency of light, *ε*_0_ is the vacuum permittivity, *P* is the normalized power density in the cross-section, *L*_NRPS_ is the length of the MO waveguide, and the *E_x_* field distribution diagram is shown in (2) of Figure 1a. According to this equation, for a given material, the NRPS in the TE mode depends on the gradient distribution of the *E_x_* component along the *x*-direction in the *y*-0-*x* waveguide section, which is achieved through the asymmetry in the in-plane distribution of the magneto-optical material.

By adjusting the phase difference ∆φ between the two waveguide arms using the RPS waveguide and the MO waveguide, unidirectional light transmission is achieved. When light propagates along the positive *z*-direction, the RPS waveguide induces a phase shift ${∆\varphi }_{RPS}=\beta \times ∆L_{RPS}=\pi /2+2m\pi$ (where *m* is an integer and β is the propagation constant of the reciprocal waveguide), while the two MO waveguide arms introduce a phase shift of ${∆\varphi }_{NRPS}=∆\beta \times L_{NRPS}=-\pi /2$. The total phase difference then becomes ∆φ=2mπ, which satisfies constructive interference, allowing light to be efficiently coupled into the output waveguide with low coupling loss and transmitted through the device, as shown in Figure 1b. When light propagates along the negative *z*-direction, the RPS waveguide still induces a phase shift ${∆\varphi }_{RPS}=\beta \times ∆L_{RPS}=\pi /2+2m\pi$, but due to the MO NRPS effect, the two MO waveguide arms introduce a phase shift ${∆\varphi }_{NRPS}=∆\beta \times L_{NRPS}=\pi /2$. The total phase difference becomes ∆φ=(2m+1)π, resulting in destructive interference, causing poor coupling with the input waveguide mode and the majority of the light dissipating as radiation, thereby achieving optical isolation, as shown in Figure 1c. By precisely adjusting the length of the MO waveguide and the length difference between the two waveguide arms, the optimal phase difference can be achieved, further optimizing the performance of the device.

## 3. Results and Discussion

### 3.1. Optimization of MO Waveguide Parameters and NRPS Calculation

Based on the MO waveguide materials involved in Figure 1b, the dielectric tensor of the MO material is defined according to Equation (1). On this basis, the field distribution of the corresponding modes in the waveguide at a wavelength of 1550 nm was calculated using FEM. The parameters obtained from the mode field calculation include the effective refractive index $n_{eff}$ (where $n_{eff}=\beta /k_{0}$, and *k*_0_ is the wavenumber in the vacuum), along with *β_f_* and *β_b_*, and ∆β, etc. In the simulation, the initial length of the MO waveguide was set to 3 μm, and the substrate thickness was 2 μm (to ensure the stability of the magnetic field distribution). The waveguide structure mesh consists of 396,560 domain elements, 53,236 boundary elements, and 1678 edge elements, with a total of 139,501 elements, and the convergence error reached 2.6 × 10^−9^. The dense mesh allows for a more accurate description of the spatial distribution of the magnetic and optical fields, capturing the gradient variations of the magnetic field, thereby better simulating the polarization rotation effect and the isolator performance.

**Figure 2:**
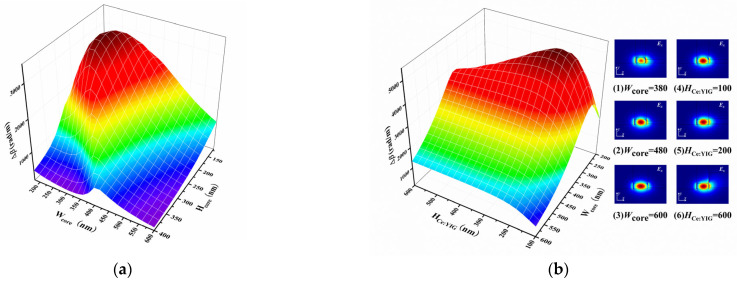

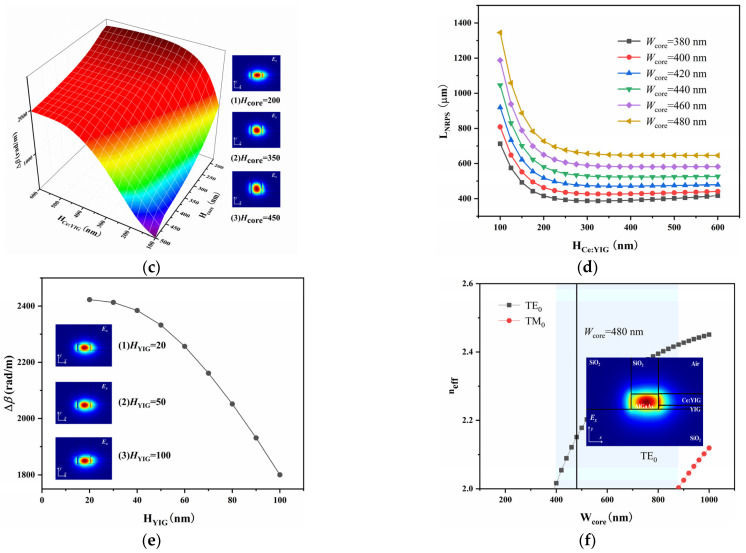
(**a**) The functional relationship between Δ*β* and the thickness and width of the core layer. (**b**) Δ*β* with respect to different *W*_core_ and *H*_Ce:YIG_ variations. (**1**–**3**) show the *E_x_* field distribution along the *x*-direction when *W*_core_ = 380, 480, or 600 nm, respectively. (**4**–**6**) show the *E_x_* field distribution for *H*_Ce:YIG_ = 100, 200, or 600 nm, respectively. (**c**) Δ*β* with respect to different height combinations. (**1**–**3**) show the *E_x_* field distribution along the *x*-direction when *H*_core_ = 200, 350, or 450 nm, respectively. (**d**) The relationship between *L*_NRPS_ and *H*_Ce:YIG_. (**e**) The dependence of Δ*β* on *H*_YIG_. (**1**–**3**) show the *E_x_* field distribution along the *x*-direction when *H*_YIG_ = 20, 50, or 100 nm, respectively. (**f**) The effective index distribution of the waveguide modes. The light blue area indicates the region where the waveguide satisfies the TE_0_ single-mode condition. The solid black line represents the TE_0_ present in the waveguide when *W*_core_ = 480 nm.

The trend in Δ*β* with changes in the core layer thickness and width is shown in Figure 2a. In the simulation, the initial settings are as follows: *H*_Ce:YIG_ = 100 nm and *H*_YIG_ = 50 nm. When the *H*_core_ is fixed, the *W*_core_ first increases and then decreases; when the *W*_core_ is fixed, the thickness variation follows a similar trend. In this simulation, *H*_core_ = 220 nm (consistent with the standard SOI substrate) was selected, and the relationship between Δ*β*, *W*_core_, and *H*_Ce:YIG_ was calculated. As shown in Figure 2b, as *W*_core_ increases, the change gradient of *H*_Ce:YIG_ becomes more gradual. At the same time, as *H*_Ce:YIG_ increases, *W*_core_ consistently increases at first and then decreases. When *H*_Ce:YIG_ = 100 nm, the distribution of the *E_x_* field along the *x*-direction in the TE_0_ mode was calculated, as shown in (1)–(3) in Figure 2b. As *W*_core_ increases, the TE mode becomes primarily concentrated in the AlGaAs core layer, reducing the energy coupled into the MO material layer. This leads to a decrease in the *E_x_* field gradient in the MO material layer, a smaller integration region, and consequently a reduction in Δ*β*. Additionally, for *W*_core_ = 480 nm, the *E_x_* field distribution was considered, as shown in (4)–(6) in Figure 2b. As *H*_Ce:YIG_ increases, the energy coupled into the MO material layer increases, the *E_x_* field gradient becomes stronger, and the integration region expands, resulting in an increase in Δ*β*. After *H*_Ce:YIG_ = 200 nm, the *E_x_* field is completely confined within the AlGaAs/MO layer, and no significant changes occur.

Subsequently, the variation in Δ*β* in the AlGaAs/Ce:YIG waveguide with different height combinations was calculated when *W*_core_ = 480 nm. As shown in Figure 2c, when *H*_Ce:YIG_ is constant, the change in Δ*β* is relatively small as *H*_core_ increases. However, when *H*_Ce:YIG_ is constant, Δ*β* rapidly tends to stabilize. The variation in the *E_x_* field distribution with *H*_core_ (*H*_Ce:YIG_ = 100 nm) is also considered here, as shown in (1)–(3) in Figure 2c. Similar to the change in *W*_core_, as the core thickness increases, the mode becomes primarily concentrated in the core layer, resulting in a decrease in Δ*β*. The above analysis indicates that in the TE_0_ mode, Δ*β* is not affected by the gradient of the *E_x_* component along the *y*-direction in the MO material layer, but only by the gradient in the *x*-direction. Therefore, we further considered the relationship between *L*_NRPS_ and *H*_Ce:YIG_ when *W*_core_ varies between 380 nm and 480 nm with a step size of 20 nm. As *W*_core_ increases, *L*_NRPS_ increases. For each width, *L*_NRPS_ decreases first and then stabilizes, as shown in Figure 2d. This is because, as explained earlier, as *H*_Ce:YIG_ increases, the energy coupled into the MO material layer increases, leading to an increase in Δ*β*. According to the formula $L_{NRPS}=-\pi /2/∆\beta$, when the Δ*β* increases, the wavelength *L*_NRPS_ becomes shorter, and vice versa. After optimizing these parameters, we selected *H*_Ce:YIG_ = 175 nm, *W*_core_ = 480 nm, and *H*_core_ = 220 nm, with an NRPS of 2332.622 rad/m and a waveguide length of 673.40 μm, which is 451.60 μm shorter than the current SOI structure [18].

To address the lattice mismatch between the Ce:YIG and the substrate, a YIG seed layer was used as an isolation layer. Figure 2e shows the dependence of Δ*β* on *H*_YIG_. As *H*_YIG_ increases, Δ*β* decreases. This is because as the thickness of *H*_YIG_ increases, the energy coupled into Ce:YIG decreases, as shown in (1)–(3) in Figure 2e. The thickness of the YIG seed layer was chosen to be 50 nm, which ensures the crystallization quality of the Ce:YIG while preventing the seed layer from being too thick, which could cause a large number of waveguide modes to fail to couple into the Ce:YIG material layer. The waveguide of the NRPS must meet the single-mode condition for proper interferometer operation to avoid unnecessary TE-TM mode conversion and ensure precise phase control. According to the results in Figure 2f, when *W*_core_ is in the range of 400–880 nm (as indicated by the light blue region), *W*_core_ = 480 nm satisfies the single-mode condition for the TE_0_ mode. Within a certain range of core widths, the waveguide can effectively suppress higher-order modes, thus maintaining the purity and stability of the signal.

### 3.2. Reciprocal Waveguide Structure and MMI Couplers Design

**Figure 3:**
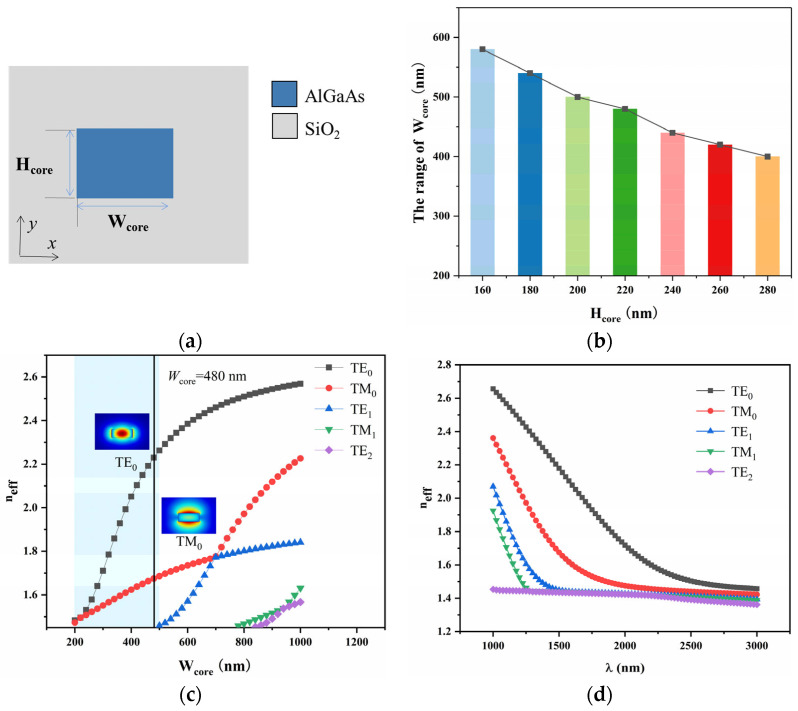
(**a**) Surrounded by SiO_2_ with reciprocal waveguide structure. (**b**) Relationship between width range and thickness variation. (**c**) Effective refractive index distribution of waveguide modes as function of waveguide width. Solid black line represents modes present in waveguide when *W*_core_ = 480 nm. (**d**) Dispersion curves as function of wavelength *λ*.

The difference in arm lengths of the reciprocal waveguide provides a phase difference of +π/2 or 2mπ + π/2 for light traveling in the forward and reverse directions. The reciprocal waveguide structure is the same as the MO waveguide, as shown in Figure 3a. The AlGaAs waveguide is surrounded by SiO_2_, with a core width of *W*_core_ = 480 nm and a core thickness of *H*_core_ = 220 nm. Using FEM calculations, the propagation constant was found to be 8,630,128.944 rad/cm, and the reciprocal phase shift of π/2 occurred with a $∆L_{RPS}=\left(\pi /2+2m\pi \right)/\beta =$182 nm (*m* = 0), which resulted in a very small fabrication tolerance for the actual device. Therefore, the arm length differences required to generate reciprocal phase shifts of 10.5π, 20.5π, 30.5π, 40.5π, 50.5π, and 60.5π were calculated, as shown in Table 1. In the simulation, the full mesh includes 512,640 domain elements, 60,144 boundary elements, and 1048 edge elements, with a total of 180,237 elements, and the convergence error is 5.9 × 10^−7^.

To ensure single-mode operation of the device, we calculated the dependence of *H*_core_ (160–280 nm) and *W*_core_ range variations. Figure 3b shows that as *H*_core_ increases, the allowable range of *W*_core_ gradually decreases. When *H*_core_ = 220 nm, *W*_core_ in the range of 200–480 nm satisfies the single-mode condition for the TE_0_ mode. Additionally, we considered the effective refractive index profiles of the first five waveguide modes for this structure, as shown in Figure 3c. The figure shows the electric field distributions for the TE_0_ and TM_0_ modes that can be transmitted in this structure. Figure 3d displays the dispersion curves for the first five modes as a function of wavelength. As shown in the figure, when the effective refractive index *n_eff_* > 1.45 (the *n_eff_* of the cladding; cutoff value), the mode can propagate; when *n_eff_* < 1.45, the mode cannot propagate.

The MMI coupler offers low loss, wide optical bandwidth, and high thermal stability, with insensitivity to manufacturing tolerances [34]. It has a simple structure, is easy to integrate, efficiently distributes optical power, accommodates different modes, and features a compact design with a low cost. It is widely used in integrated optics, communications, and sensor applications. The 3 dB coupler is surrounded by SiO_2_. To optimize the performance of the MMI coupler, the width of the multimode interference region *W*_coupler_ was set to 6 μm to avoid excessive insertion loss and minimal crosstalk. The coupling length *L*_coupler_ is as follows:

(4) $$L_{coupler}=\frac{3\pi }{{8(\beta }_{0}-\beta_{1})}$$

**Figure 4:**
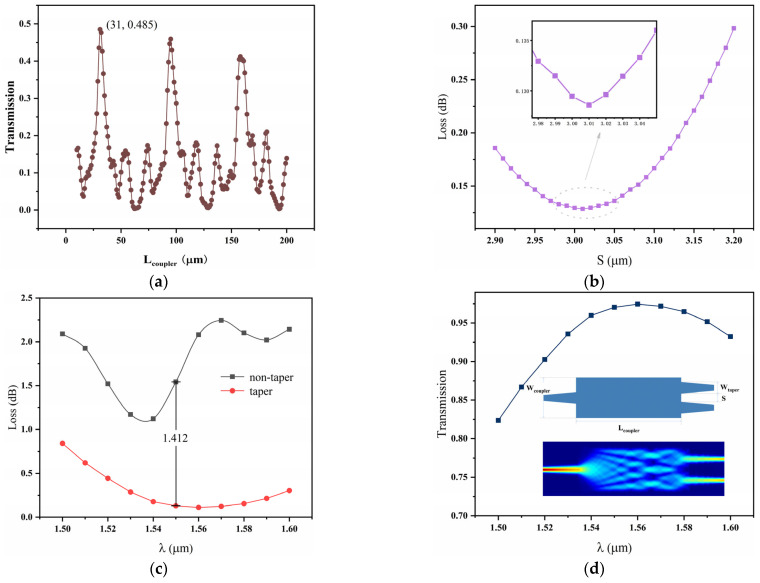
(**a**) The function of the transmission and coupling region length *L*_coupler_ and its maximum transmittance. (**b**) The dependence between the gap between the output waveguides (*S*) and insertion loss. (**c**) The impact of tapered waveguides on loss with and without tapering in the wavelength range *λ* of 1500–1600 nm. (**d**) Transmittance in the *λ* range of 1500~1600 nm and the mode field distribution of the MMI coupler.

The propagation constants *β*_0_ and *β*_1_ are the values for TE_0_ and TE_1_ modes, respectively, which are 10,348,032.630 and 10,308,387.791 rad/m. Using the formula, the coupling length *L*_coupler_ = 29.72 μm is obtained. The coupling length *L*_coupler_ was optimized using the EME method, with the results shown in Figure 4a. When *L*_coupler_ = 31 μm, the maximum transmission of 0.485 is achieved (for a single output waveguide). Next, the relationship between the gap *S* between the two output waveguides of the AlGaAs waveguide-based coupler and insertion loss was calculated. Figure 4b shows that the insertion loss is minimized when *S* = 3.01 μm. By using a linear taper on the input/output waveguides, the waveguide width (480 nm) can gradually transition, reducing reflection and loss caused by a mode mismatch, thereby minimizing losses and improving the system’s optical signal transmission efficiency. In Figure 4c, when the taper width *W*_taper_ = 1.30 μm, the insertion loss in the straight waveguide configuration increases by 1.412 dB, with the final loss being 0.130 dB. Finally, the transmission of the coupler in the wavelength range of 1500–1600 nm was calculated. The transmission at 1550 nm is 0.97 dB, and the mode field distribution of the final structure is shown in Figure 4d.

### 3.3. Insertion Loss, Isolation Bandwidth, and Tolerance

In the performance evaluation of optical devices, insertion loss, isolation bandwidth, and manufacturing tolerance are key indicators that affect the device’s efficiency, reliability, and long-term stability. First, insertion loss is the difference in signal strength between the light signal entering and leaving the device, typically expressed in decibels (dB). It represents the energy loss of the signal as it passes through the device. Lower insertion loss means that most of the optical power can be effectively transmitted without excessive attenuation. The losses in the designed MZI-type magneto-optical isolator come from the following parts: the MMI coupler, bent waveguide, coupling between the RPS waveguide and the MO waveguide, and the MO waveguide itself.

**Figure 5:**
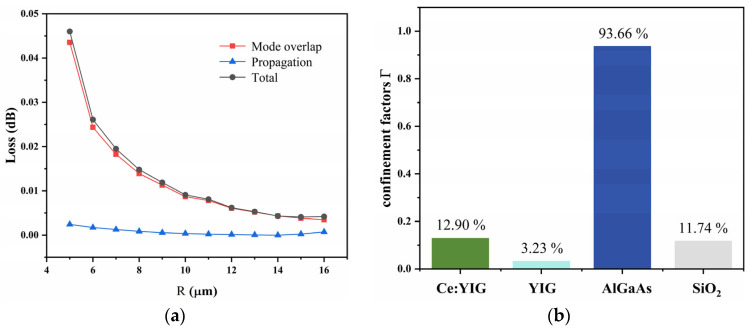
(**a**) Relationship between loss and bending radius *R*. (**b**) Confinement factors of each layer in MO waveguide.

In the previous section, the structural parameters of the MMI coupler were optimized, and a coupler with a tapered waveguide was used, achieving a loss as low as 0.13 dB (a single coupler). The loss in the bent waveguide mainly consists of two parts: the propagation loss of the TE_0_ mode and the mode overlap loss between the bent waveguide and the straight waveguide. A smaller bending radius causes severe mode mismatch at the bend, resulting in part of the optical energy being “scattered” and causing signal loss. To reduce the loss of the bent waveguide, the bending radius typically needs to be optimized. Therefore, an FDE solver was used to calculate the relationship between the bending radius *R* and the loss, as shown in Figure 5a. The waveguide bending angle was set to π/2, and the orientation angle was set to zero (i.e., the bent waveguide and the straight waveguide are in the same plane). As the radius *R* increases, the loss rapidly decreases and tends to stabilize, with the loss mainly coming from the overlap loss. When *R* is 15 μm, the transmission loss and overlap loss are 0.00024 dB and 0.00382 dB, respectively, and the total loss of the eight bent waveguides is 0.03 dB. Additionally, considering the coupling loss between the RPS waveguide and the MO waveguide, the total coupling loss was calculated to be 0.61 dB using the FDE method.

Finally, the propagation loss of the MO waveguide is discussed, with the main cause being material absorption. In the TE mode, the equation for calculating the loss is as follows:

(5) $$\alpha_{MO}=\alpha_{Ce:YIG}\times \Gamma_{Ce:YIG}+\alpha_{YIG}\times \Gamma_{YIG}+\alpha_{AlGaAs}\times \Gamma_{AlGaAs}+\alpha_{{SiO}_{2}}\times \Gamma_{{SiO}_{2}}$$

(6) $$\Gamma =\frac{nc_{0}\varepsilon_{0}\iint \left|E^{2}\right|dxdy}{\iint_{\infty }Re(E\times H^{*})\cdot \tilde{z}\cdot dxdy}$$

where $\alpha_{Ce:YIG}$, $\alpha_{YIG}$, $\alpha_{AlGaAs}$, and $\alpha_{{SiO}_{2}}$ represent the losses of each layer, while $\Gamma_{Ce:YIG}$, $\Gamma_{YIG}$, $\Gamma_{AlGaAs}$, and $\Gamma_{{SiO}_{2}}$ correspond to the loss confinement factors of the multilayer waveguide. *c*_0_ is the speed of light in a vacuum, and *ε*_0_ is the permittivity of free space. Specifically, the propagation loss of the RPS waveguide is 0.4 dB/cm [26], while the loss of the magnetic material Ce:YIG is 50 dB/cm [31]. Using the FEM, the loss confinement factors for Ce:YIG, YIG, AlGaAs, and SiO₂ were calculated, and the results are shown in Figure 5b. In the calculation, the cross-sectional structure of the MO waveguide is shown in Figure 2f, with the core layer thickness and width being 220 nm and 480 nm, respectively, and the thicknesses of Ce:YIG and YIG being 175 nm and 50 nm, respectively. At the same time, the thickness of the substrate and cover layer is set to 2 μm. The mesh setup is the same as that for the MO waveguide. The calculated confinement factors for the materials are as follows: Ce:YIG = 12.9%, YIG = 3.23%, AlGaAs = 93.66%, and SiO₂ = 11.74%. Since the loss of SiO₂ is typically very low, its contribution to the total loss can be neglected, so the loss from $\alpha_{{SiO}_{2}}\times \Gamma_{{SiO}_{2}}$ is approximately 0 dB. After optimization, the length of the MO waveguide is 673.40 μm, and the loss of the MO waveguide is 0.57 dB. Finally, the total loss of the entire device is 1.47 dB, with the losses of each part detailed in Table 2.

FoM is defined as the ratio of the NRPS induced by the MO effect to the TE_0_ mode propagation loss: $FoM=\beta /\alpha_{MO}$. When the propagation constant β is 2332.622 rad/m, the FoM is 2.76 rad/dB. Compared to the existing report [8], this device exhibits a larger FoM, indicating that the MO waveguide has strong nonreciprocal behavior, low loss, and high bandwidth, achieving an excellent balance between efficient isolation and low-loss transmission.

**Figure 6:**
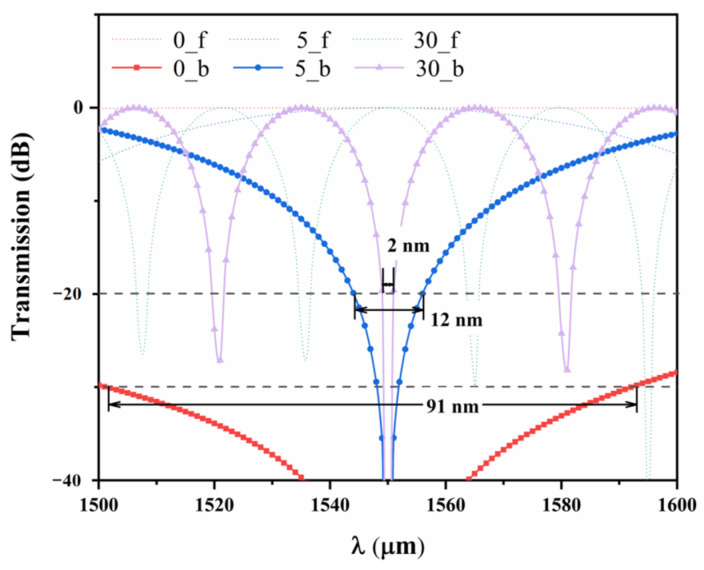
The transmission spectra of the optical isolator in the forward and backward propagation directions with different values of *m*.

The isolation bandwidth defines the wavelength range within which the device can effectively isolate reverse-propagating light signals. A wider isolation bandwidth ensures that the device provides stable signal isolation over a broader wavelength range, preventing interference from reverse signals. Based on the FEM method, this optical isolator has high isolation and wide bandwidth in the wavelength range of 1500 to 1600 nm, as shown in Figure 6. When *m* = 0, the device achieves a wide bandwidth of up to 91 nm at 30 dB. However, when *m* = 30, the bandwidth at 20 dB is only 2 nm. Compared to the previous TM mode study [27], although the isolation of the TE-mode isolator has decreased by 5 dB, improvements have been made in bandwidth and loss. Specifically, the bandwidth exceeds 91 nm, with the loss controlled within 1.47 dB, achieving a high-quality performance standard, as shown in Table 3. Moreover, the performance of the isolator in this study is also superior to previous TE-mode optical isolators.

As the arm length difference increases, both the operating bandwidth and isolation decrease. This is because the propagation path difference between the two arms becomes larger, increasing the periodic variation of interference and making the device more wavelength-dependent. This length difference is achieved by using RPS waveguides of different lengths in the upper and lower layers (asymmetric MZI type). During the manufacturing process of the optical isolator, inevitable small errors can indeed have a significant impact on the phase-matching capability of the device. This impact not only affects its performance but can also lead to system instability, efficiency degradation, or even failure. Therefore, we analyzed the manufacturing tolerances of the device, as shown in Table 4. Unlike the TM mode isolator, the TE mode is most sensitive to width, with a bandwidth of only ±6 nm. The manufacturing tolerance for LNRPS is relatively large, at ±27.11 μm.

## 4. Conclusions

In summary, this paper presents a TE-mode MO isolator based on the AlGaAs-on-insulator platform, and describes the optimization of the MO waveguide structure using the FEM method. The core layer thickness was determined to be *H*_core_ = 220 nm, core width *W*_core_ = 480 nm, Ce:YIG layer thickness *H*_Ce:YIG_ = 175 nm, and YIG layer thickness *H*_YIG_ = 50 nm. With this structure, the Δ*β* value was 2332.62 rad/m, and the corresponding waveguide length was 673.40 μm. Based on the optimized MO structure, MMI couplers and RPS waveguides were designed. For the MMI coupler, the loss was reduced by 1.41 dB when the tapered waveguide width was set to 1.3 μm. A loss analysis of the overall device was conducted, and the total loss was reduced to 1.47 dB when the bend radius was 15 μm. The device achieved a high FoM value of 2.76 rad/dB and a wide operating bandwidth of 91 nm at 30 dB isolation. The manufacturing tolerance analysis revealed that the device was most sensitive to changes in *W*_core_, with a tolerance of only ±6 nm, indicating the strong sensitivity of the TE mode to the gradient distribution of the *E_x_* component in the *x*-direction. This study provides an optimized design for TE-mode isolators based on the AlGaAs-on-insulator platform, offering low loss, high isolation, wide bandwidth, and good manufacturing tolerance adaptability. These advantages make the proposed device well suited for nonlinear optical platforms, particularly in efficient and stable integrated photonic systems.

## Figures and Tables

**Table 1:** The difference in length of the reciprocal waveguide arm under different *m* of the isolator.

*m*	Δ*L* (nm)
0	182.01
5	3822.27
10	7462.53
15	11,102.79
20	14,743.05
25	18,383.31
30	22,023.56

**Table 2:** The losses of each part of the magneto-optical isolator in the TE mode.

Device Sections	Loss (dB)
MMI coupler	0.26
bent waveguide	0.03
coupling between RPS waveguide and MO waveguide	0.61
MO waveguide	0.57
total loss	1.47

**Table 3:** Performance of broadband TE optical isolators.

**Device Type**	**Polarization**	**Isolation Ratio** **(dB)**	**Bandwidth** **(nm)**	**Losses** **(dB)**	**FoM** **(rad/dB)**	
SOI-MZI	TE	26.7	/	33.4	/	Ref. [35]
SOI-MZI	TE	30	2	9	/	Ref. [18]
SOI-ring	TE	25	40	6.5	/	Ref. [36]
SOI-ring	TE	20	/	11.5	/	Ref. [18]
SOI-ring	TE	22	/	4.3	/	Ref. [37]
GaAs-MZI	TM	35	53.5/70	2.59/2.25	/	Ref. [27]
AlGaAs-MZI	TE	30	91	1.47	2.76	This work

**Table 4:** The geometric tolerances of the proposed MZI isolators.

Device Geometries	Value	Tolerances
*H*_core_	220 nm	±12 nm
*W*_core_	480 nm	±6 nm
*H*_Ce:YIG_	175 nm	±10 nm
*L*_NRPS_	673.40 µm	±27.11 µm
Δ*L*_RPS_	182.01 nm	±7.32 nm

## Data Availability

Data are contained within the article.
